# Psychogenic and Organic Amnesia. A Multidimensional Assessment of Clinical, Neuroradiological, Neuropsychological and Psychopathological Features

**DOI:** 10.1155/2007/193140

**Published:** 2007-02-12

**Authors:** Laura Serra, Lucia Fadda, Ivana Buccione, Carlo Caltagirone, Giovanni A. Carlesimo

**Affiliations:** ^1^Fondazione IRCCS Santa LuciaRomaItaly; ^2^Clinica NeurologicaUniversità Tor VergataRomaItaly

**Keywords:** Amnesia, psychogenic origin, organic origin

## Abstract

Psychogenic amnesia is a complex disorder characterised by a wide variety of symptoms. Consequently, in a number of cases it is difficult distinguish it from organic memory impairment. The present study reports a new case of global psychogenic amnesia compared with two patients with amnesia underlain by organic brain damage. Our aim was to identify features useful for distinguishing between psychogenic and organic forms of memory impairment. The findings show the usefulness of a multidimensional evaluation of clinical, neuroradiological, neuropsychological and psychopathological aspects, to provide convergent findings useful for differentiating the two forms of memory disorder.

## 1. Introduction

*Psychogenic* or *dissociative amnesia* (DSM-IV-TR) [1] is a clinical syndrome characterised by a memory disorder of nonorganic origin. Following Kopelman [31,33], psychogenic amnesia can either be situation specific or global. *Situation specific amnesia* refers to memory loss for a particular incident or part of an incident and can arise in a variety of circumstances, resulting in post-traumatic stress disorder (e.g., committing an offence, being a victim of an offence or of child abuse). Instead *Global psychogenic amnesia*, also called Fugue State [21], is characterised by a sudden loss of autobiographical memories generally going back many years and frequently associated with a loss of personal identity – that is, knowledge and consciousness of the self – and a period of wandering. According to Kopelman [33], there are three main predisposing factors for global psychogenic amnesia: *i*) a history of transient, organic amnesia due to epilepsy [52], head injury [4] or alcoholic blackouts [20]; *ii*) a history of psychiatric disorders such as depressed mood, and *iii*) a severe precipitating stress, such as marital or emotional discord [23], bereavement [49], financial problems [23] or war [21,48]. Apparently, patients who have previously experienced transient organic amnesia and then become depressed are particularly likely to develop psychogenic amnesia if faced with severe precipitating stress [33].

Although non organic memory impairment may be suspected in psychogenic amnesics based on the qualitative features of their memory loss (which differ from those typically seen in organic amnesia [36]), on its abrupt onset generally related to psychological distress [39] and on a lack of clinical and instrumental evidence of damage to cerebral regions known to be involved in memory functioning, in a number of

ISSN 0953-4180/07/$17.00 © 2007 – IOS Press and the authors. All rights reserved cases the great variability among patients raises perplexities about the exact nature of the memory deficit. Table 1 reports some of the clinical, neuroradiological and psychopathological findings in a selection of cases of global psychogenic amnesia reported in the literature in the last decade. In most cases a mild head trauma was the precipitating event. However, cases were reported in which no precipitating event could be found. Neuroradiological (MRI or TC) and perfusional (PET or SPECT) exams were generally negative for organic brain damage; thus, when evidence of organic damage was found it was generally interpreted as incidental. However, Markowitsch [41] suggested that in some cases of psychogenic amnesia the presence of reduced right frontotemporal perfusion might be the functional basis of the autobiographical memory impairment. Finally, a history of psychiatric disease and an emotionally stressful event precipitating the amnesic status were reported in most, but not in all, cases. Considering the particular nature of this syndrome, it is surprising that only a few studies have assessed the personality profile of patients using ad hoc instruments.

The neuropsychological profile of the memory impairment, which was widely investigated in some cases, is controversial (see Table 2). Indeed, while the impairment of retrograde autobiographical memory is the cognitive hallmark of the syndrome (but see [28] for a case of psychogenic amnesia without a retrograde deficit), anterograde memory was normal in some cases and affected in others. Finally, short-term, semantic and implicit memories, when investigated, were mostly normal. In many patients the presence of a selective deficit of retrograde memory (e.g. [16,25,30]) raised a question about the relationship between psychogenic amnesia and the picture of focal retrograde amnesia (a condition characterised by a disorder in the retrieval of premorbid events and information in which new learning is unaffected [24]). In a review of the literature, Kopelman [32] distinguished cases of focal retrograde amnesia in which an organic origin of the disturbance could be found from those in which it could not. In the first group he included cases in which a pure retrograde deficit represented an evolution from an early anterograde/retrograde amnesic syndrome [6], cases of patients with Semantic Dementia [17] and, finally, cases with transient epileptic amnesia in which the memory gap involved only ictal activity [27]. In the second group Kopelman included patients in which focal retrograde amnesia was more likely psychogenic in nature, mainly because of a lack of clinical and/or neuroradiological evidence of organic brain damage. In Kopelman's view "although it is difficult to prove psychological causation, the logical difficulties in attributing causation where brain lesions are either very subtle or multiple have been considerably underestimated in the neuropsychological literature" [32, p. 585].

Here we present a comparison of clinical, neuroradiological, neuropsychological and psychopathological findings in a new case of global psychogenic amnesia and in two patients with amnesia underlain by organic brain damage. Our aim was to outline differential features useful for discriminating psychogenic from organic forms of memory impairment. We diagnosed our patient's amnesic syndrome as psychogenic based on the absence of any anamnestic or neuroradiological evidence of brain damage underlying the memory disorder. From the reports in the literature we expected that our psychogenic patient would differ from the two organic amnesic patients due to the following: a history of mild head trauma precipitating the amnesic syndrome, psychiatric disorder and/or emotional stress in the period preceding the onset of amnesia, and loss of personal identity in the early phases of the syndrome. We were particularly interested in the qualitative characterisation of the memory loss in our psychogenic amnesic because we wished to highlight the neuropsychological indexes differentiating organic from psychogenic forms of amnesia. In this regard, we expected that the psychogenic patient's memory impairment would be characterised mainly by a retrograde autobiographical deficit. One difference between patients with psychogenic and organic amnesia is the temporal gradient of the retrograde memory impairment. Indeed, in a typical patient with organic brain damage the retrograde memory deficit is particularly severe for the most recent premorbid period [26] (but see [10] for contrasting data on this issue), while a substantially flat temporal gradient, indicative of a homogeneous loss of retrograde memories across the entire period examined, is reported in most patients with psychogenic amnesia (e.g. [3,30]). Predictions were particularly uncertain regarding short-term and semantic memory. In fact, as reported in Table 2, in psychogenic amnesic patients both normal and impaired performances were reported in tests investigating these memory functions. Consistent with the above-reported literature, implicit memory was more likely to be spared. Finally, the available literature is particularly controversial regarding declarative anterograde memory (see Table 2). However, since a disturbance in the declarative anterograde domain is the cognitive hallmark of organic amnesia, we tried to analyse this memory component in greater

Table 1
Clinical and neuroradiological data in a selection of cases of psychogenic amnesia from the literature

| Study | History of psychiatric disease | Recent emotional stress | Precipitating event | Loss of personal identity | Neuroradiological investigation | Perfusional investigation |
|---|---|---|---|---|---|---|
| De Renzi et al. [16] | Absent | Absent | Mild head injury | Present | Normal | Normal |
| Kopelman et al. [31] | Present | Present | No | Present | Modest left ventricle enlargement | |
| Kapur [25] | Present | Present | Mild head injury | Absent | Normal | |
| Mackenzie Ross [36] | Present | Present | Mild head injury | Present | Non-specific white matter abnormalities | |
| Kessler et al. [28] | Absent | | No | Absent | Normal | Normal |
| Stracciari et al. [53] | | | | | | |
| *Case 1* | Absent | Absent | Mild head injury | Absent | Normal | Hypoperfusion on the left frontal region |
| *Case 2* | Absent | Absent | Mild head injury | Absent | Normal | Modest left parietal abnormalities |
| Markowitsch et al. [38] | Absent | Present | No | | Normal | Normal |
| Markowitsch et al. [40] | | Present | No | Present | Normal | Hypometabolism on the right temporo-frontal region |
| Barbarotto et al. [3] | Absent | | Mild head injury | Absent | Normal | Normal |
| Dalla Barba et al. [14] | Absent | Absent | No | Present | Normal | Normal |
| Izumi et al. [22] | | Present | No | Absent | Normal | |

Table 2
Neuropsychological data in selected cases of psychogenic amnesia in the literature

| Study | General intellectual efficiency | Retrograde memory | Anterograde memory | Short-term memory | Semantic memory | Procedural memory |
|---|---|---|---|---|---|---|
| De Renzi et al. [16] | Normal | Compromised | Normal | Normal | Compromised | Normal |
| Kopelman et al. [31] | Normal | Compromised | Normal | | Normal | Normal |
| Kapur [25] | Normal | Compromised | Normal | | Normal | |
| Mackenzie Ross [36] | Compromised | Compromised | Compromised | Compromised | Compromised | |
| Kessler et al. [28] | Normal | Normal | Compromised | Normal | Normal | Normal |
| Stracciari et al. [53] | | | | | | |
| *Case* | | Compromised | Normal | Normal | Normal | |
| *Case 2* | | Compromised | Normal | Normal | | |
| Markowitsch et al. [38] | Normal | Compromised | Compromised | Compromised | Normal | Normal |
| Markowitsch et al. [40] | Normal | Compromised | Normal | | Normal | |
| Barbarotto et al. [3] | Normal | Compromised | Normal | Compromised | Normal | Normal |
| Dalla Barba et al. [14] | Normal | Compromised | Normal | | Normal | |
| Izumi et al. [22] | Compromised | Normal | Compromised | Normal | | Normal |

detail. Finally, we submitted our three patients to a detailed psychodiagnostic examination to outline personality profiles able to differentiate patients whose memory disorder is organic or psychogenic in nature.

## 2. Case report

### *2.1. Psychogenic amnesia patient*

BC, a right-handed woman with 8 years of formal education, was 34 years old in 1992 when she sustained a mild head injury in the left fronto-parietal region in a car accident. She lost consciousness for 15 minutes and when she awoke appeared confused, was unable to move her limbs and experienced global amnesia for all events of her previous life and a loss of personal identity. Indeed, she could not provide her own name, address or any information about her family, including her parents, husband and son. Moreover, she could not read or write. Neuroradiological exams performed at that time were negative for focal or diffuse brain damage. Her psychiatric history was mute, with no evidence of psychotic disease or depression. No recent emotionally stressful event, which might have precipitated the psychogenic amnesia, could be found. After six months BC's motor symptoms had resolved but her recovery of retrograde memories was very partial, limited to "isles of memory" concerned with very significant autobiographical events. At that time she also showed impairment in managing daily living activities, such as cooking, ironing, and taking care of her son. New neuroradiological and neurophysiological exams (MRI and EEG), performed in 1998, were also negative for focal or diffuse brain damage; however, a neuropsychological examination showed severe impairment of short- and long-term memory in both verbal and visual-spatial domains, which contrasted with substantial sparing of general intelligence and executive functioning.

BC came under our observation in 2000 for the persistence of a pervasive impairment in remembering day-to-day events that also involved very important aspects of her life (e.g., she reported not remembering the birth of her daughter in 1994). Her memory of events that occurred or of information that was acquired before the trauma was also severely compromised. At the clinical examination BC's emotional state was very flat and she showed very low affective participation in her state. Neurological examination revealed no sign of focal motor or sensory disturbance.

### 2.2. Organic amnesia patients

BC's clinical, behavioural and neuropsychological features were compared to those of two patients with amnesia of undoubtedly organic origin. Since the memory disorder in these two patients originated from brain lesions in different areas (the basal forebrain in the first case, the mesial temporal lobe in the second), they represented a plausible (even though not homogeneous) bench mark for interpreting the qualitative pattern of BC's memory impairment.

PA was a 47-year-old right-handed woman with 17 years of formal education when, in 2000, an MRI of the brain, made to assess a persistent cephalea associated with vertigo, revealed a craniopharingioma partially occupying the third ventricle. The mass was partially removed in a neurosurgical intervention, and on subsequent days the patient was submitted to ventriculostomy for hydrocephalus. PA came under our observation in 2002. At that time she was a fully alert and co-operative individual and her neurological examination was unremarkable. Her main complaint was a pervasive impairment of memory for day-to-day events that interfered severely with her ability to return to work at the same level as before surgery.

MC, a right-handed man with 13 years of formal education, affected by insulin dependent diabetes, was 48 years old when, in 1998, he suffered a hypoglycaemic coma due to an insulin overdose. We first observed MC in February 1999. At that time he was a fully alert and co-operative individual, and his neurological examination was unremarkable. His main complaint was a severe impairment in retaining day-to-day events and information. Remote memories regarding his pre-accident life, basic cultural knowledge, public events and familiar people were apparently preserved.

## 3. Neuroradiological examination

Visual inspection of BC's cerebral MRI scan (performed in 2000) did not show any evidence of focal or diffuse damage. PA's MRI scan (performed in 2003) revealed hyperintense signal in the T2 sequences localised bilaterally in the ventral region of the frontal lobes, with a prevalence on the right side. MC's MRI scan (performed in 1998) showed bilateral atrophic changes in the hippocampal regions with consensual enlargement of the temporal horns of the lateral ventricles (see Fig. 1). BC also underwent a SPECT examination of the brain, which did not reveal any perfusion abnormality.

## 4. Neuropsychological assessment

BC, PA and MC were submitted to a global cognitive assessment of general intelligence, executive functions, language and visual-constructive abilities, and to a more detailed evaluation of memory functioning, including the assessment of short-term memory, declarative anterograde memory, implicit memory and retrograde memory for autobiographical data and semantic information. In most cases, the patients' performances on individual tests were evaluated with reference to published normative data. When norms were unavailable, or when a more qualitative analysis of performance was needed, a group of normal controls comparable to the amnesic patients for age and education was evaluated for direct comparison using the procedure of Crawford and Garthwaite [12].

### 4.1. General neuropsychological examination

The performance scores of BC, PA and MC on the neuropsychological and psychometric tests assessing general intelligence, executive functions, language and visuo-constructive abilities are reported in Table 3. BC's results on tests assessing general intelligence were inconsistent. Indeed, while her verbal, performance and full-scale IQs, as measured by the WAIS-R, were clearly abnormal her performance on Raven's Coloured Progressive Matrices was in the normal range. On

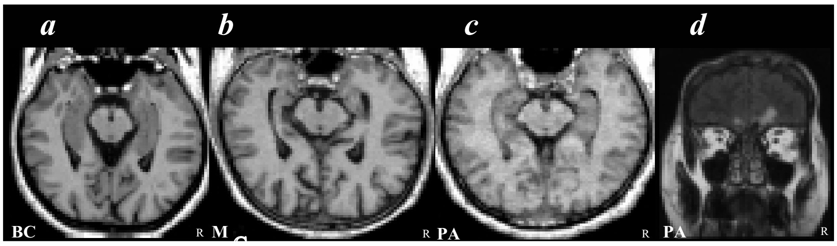

Fig. 1. Axial sections of BC's, PA's and MC's brain centred on the horizontal axis of hippocampi (panels $a$, $b$, $c$). Coronal section in T2 weighted of PA (panel $d$).

tests assessing executive functions, she was only mildly impaired on the Modified Card Sorting Test and she performed within the normal range on the Phonological Word Fluency Test. Finally, her performance on tests assessing language and visuo-constructive abilities were substantially normal.

The performances of PA and MC on tests assessing general intelligence, language and visuo-constructive skills were mostly normal. However, PA made a pathological number of perseverative errors on the Modified Card Sorting Test and performed below normal on the Word Fluency test. This is indicative of a mild deficit in executive functions and is frequently seen in diencephalic amnesia [46].

### *4.2. Memory examination*

#### *4.2.1. Short-term and anterograde episodic memory*

The performance scores of BC, PA and MC on tests of anterograde memory are reported in Table 4. As is typically observed in patients with organic amnesia, PA's and MC's memory quotients on the Wechsler Memory Scale were substantially lower than their WAIS IQs (26 and 35 points, respectively). Instead, BC's memory quotient, which was definitely low, was higher than her IQ.

In a more detailed neuropsychological assessment of anterograde memory BC was very compromised on free recall measures of episodic long-term memory involving both verbal data (immediate and delayed recall of word lists and of a short story) and visual-spatial data (immediate and delayed reproduction of Rey's Figure and multitrial learning of a supraspan sequence on the Corsi Block test). In a y/n recognition test for a word list her score was in the lower limit of the 95% tolerance interval. However, her performance was definitely abnormal on four subtests of Warrington's CAMDEN battery [54] that evaluate recognition memory for both verbal and visual-spatial data. BC also obtained pathological scores on three short-term memory tasks (digit and spatial spans and immediate recognition of individual visual items).

The performance of PA and MC on episodic memory tests was consistently below normal (with the exception of PA's learning of the supraspan spatial sequence). However, their performance scores on short-term memory tasks were in the normal range.

#### *4.2.2. Implicit memory*

BC, PA and MC were given a Stem Completion test [8] to assess their verbal repetition priming. On this test subjects are first presented visually with a list of 20 words. Immediately after they are given a list of 40 three-letter stems and are requested to complete each stem with the first word that comes to mind. What makes this an implicit memory task is that no mention is made that 20 of the 40 stems can be completed with the words of the previously studied list. The priming level (advantage in the correct completion of studied over unstudied words) was 3, 1 and 2, respectively. In no case did it significantly differ from the mean of a group of 10 normal controls ($M = 3.9$, $SD = 2.2$; $t = 0.4$, 1.3 and 0.8, respectively; p consistently $>$0.10).

BC was also administered a Mirror Reading test to evaluate procedural learning [11]. In this test subjects are asked to read aloud as fast and accurately as possible twenty mirror reversed word triads printed left to right in lower case on a sheet of A4 paper. In a 60 min. delayed trial subjects are requested to read 10 of the previously read triads and 10 new triads. The examiner uses a stopwatch to check the time needed to read each triad. We analysed the subjects' performances on repeated and not repeated stimuli separately to evaluate their ability to acquire task-related procedures and to benefit from repetition of specific stimuli. BC was 17.3% faster at reading the new triads in the 60 min. de-

Table 3
Amnesic patients' performance scores on tests of the general neuropsychological battery. With the exception of the WAIS-R, reported scores are adjusted for age, gender and years of formal education according to published normative data (references in brackets)

| | Test | BC | PA | MC | Lower limit of the 95% tolerance interval |
|---|---|---|---|---|---|
| **General Intelligence** | **WAIS-R** [56] | | | | |
| | Information | 4 | 11 | 15 | |
| | Digit span | 3 | 12 | 11 | |
| | Vocabulary | 3 | 11 | 12 | |
| | Arithmetic | 3 | 10 | 12 | |
| | Comprehension | 5 | 3 | 15 | |
| | Similarities | 2 | 7 | 16 | |
| | Picture Completion | 2 | 8 | 11 | |
| | Picture Arrangement | 5 | 10 | 10 | |
| | Block Design | 4 | 7 | 6 | |
| | Object Assembly | 9 | 9 | 6 | |
| | Digit Symbol | 2 | 4 | 8 | |
| | Verbal IQ | 59* | 101 | 128 | |
| | Performance IQ | 70* | 106 | 110 | |
| | Full-scale IQ | 60* | 103 | 122 | |
| | **Raven's Coloured Matrices** [9] | 25.5 | 26.8 | 25.3 | 18.9 |
| **Executive Function** | **Modified Card Sorting Test** [43] | | | | |
| | Criteria achieved | 3.9* | 4.9 | 5.9 | 4.2 |
| | Perseverative Errors | 6.3 | 10.3* | 0 | 7.6 |
| | **Phonological Verbal Fluency** [9] | 17.9 | 7.9* | 27.9 | 17.3 |
| **Language** | **Phrase Construction** [9] | 19.4 | 18 | 16.7 | 8.7 |
| **Constructional praxis** | **Copy of Drawings** [9] | 8.9 | 9.8 | 9.3 | 7.1 |
| | **Copy of Drawings with Landmarks** [9] | 66.8 | 64.3 | 70 | 61.8 |
| | **Rey's Figure Copy** [7] | 26.7 | 32.4 | 27.6 | 23.7 |

*Pathological performance.

layed trial ($M = 30.2$ sec.) with respect to the first trial ($M = 36.5$ sec.), a performance indicative of normal procedural learning (average rate of improvement in 22 normal controls = 14.7%; $t = 0.1$; $p = n.s.$). Instead, BC's improvement in reading the old triads during the delayed trial with respect to reading the same triplets in the first trial was only 10.5%. This performance indicated low contribution of episodic memory to the task (average rate of improvement in the normal controls = 41.6%; $t = 1.6$; $p = 0.06$).

### 4.2.3. *Retrograde memory*

BC's and PA's retrograde memory for autobiographical events was examined by means of Crovitz's procedure [13], which requires that the experimental subject generate an autobiographical event for each of 60 stimulus words proposed by the examiner. The overall score on this task, which takes into account the richness of detail of the events reported and the precision of the spatio-temporal context in which the event is inserted, was 41.5 for BC and 51.1 for PA. In both cases it was clearly below 72.82, which is the lower limit of the normal distribution [18]. As shown in Fig. 2, which reports the temporal distribution of events reported in Crovitz's test by BC and PA and by a group of 6 normal controls aged 35 to 50, performance of both amnesics was particularly poor for the 15–20 years preceding the onset of amnesia. Both patients performed relatively better for the period following the onset of amnesia. Finally, while PA's scores were within the average of normal controls for more remote periods (more than 15 years prior to amnesia), BC did not display a similar preservation of memory for autobiographical events that had occurred long before the onset of amnesia.

### 4.2.4. *Semantic memory*

BC performed poorly on the WAIS-R subtests investigating semantic competence (scaled scores on the Vocabulary and Information subtests = 3 and 4, respectively). Instead, PA and MC performed normally on both the Vocabulary (scaled scores = 11 and 12, respectively) and the Information (scaled scores = 11 and 15, respectively) subtests, demonstrating a substantial preservation of semantic knowledge.

BC was also given a Semantic Questionnaire investigating basic knowledge of living and non living things [35]. This test investigates six categories, three of living things (animals, fruit and vegetables) and three

Table 4
Amnesic patients' performance scores on tests assessing short-term and anterograde long-term memory functioning. With the exception of the WMS, reported scores are adjusted for age, gender and years of formal education according to published normative data (references in brackets)

| | Test | BC | PA | MC | Lower limit of the 95% tolerance interval |
|---|---|---|---|---|---|
| **General assessment** | **Wechsler Memory Scale** [55] | | | | |
| | MQ | 64 | 77 | 90 | |
| | IQ-MQ | −4 | 26 | 35 | |
| **Short-term memory** | | | | | |
| **Verbal** | **Digit Span forward** [45] | 3* | 5.2 | 6.5 | 3.7 |
| **Visual-spatial** | **Corsi Span forward** [45] | 3* | 5.5 | 4.5 | 3.5 |
| | **Immediate Visual Memory** [9] | 12.8 | 17.1 | 19.8 | 13.8 |
| **Anterograde long-term memory** | | | | | |
| **Verbal** | **Semantically unrelated 15-word list** [9] | | | | |
| | Immediate recall (five trials) | 20* | 12.8* | 23.1* | 28.5 |
| | Delayed recall (15 min) | 0* | 0* | 0* | 4.6 |
| | **Semantically related 16-word list** [42] | | | | |
| | Immediate recall (five trials) | 27.1* | – | 27.2* | 27.9 |
| | Delayed recall (15 min) | 2.7* | – | 0* | 4.7 |
| | Recognition (yes/no) | 22.5 | – | 18.9* | 22.5 |
| | **Short Story** [7] | | | | |
| | Immediate recall | 0* | 3.1 | 5.2 | 3.1 |
| | Delayed recall (20 min) | 0* | 0* | 0* | 2.3 |
| | **CAMDEN** [54] Word Recognition | 16* | – | – | 21 |
| **Visual-spatial** | **Rey's Figure** [7] | | | | |
| | Immediate recall | 3.1* | 0.6* | 0* | 6.4 |
| | Delayed recall (20 min) | 2.3* | 1* | 0* | 6.3 |
| | **Supraspan Learning** [51] | 0* | 8.7 | 2.3* | 5.7 |
| | **CAMDEN** [54] Pictorial Recognition | 13* | – | – | 27 |
| | **CAMDEN** [54] Topographical Recognition | 13* | – | – | 19 |
| | **CAMDEN** [54] Face Recognition | 11* | – | – | 18 |

*Pathological performance.

of nonliving things (tools, furniture and vehicles). The Questionnaire includes six questions for each of 30 items (e.g., butterfly); the first two pertain to Superordinate information [one general superordinate (e.g., Is it an animal, a vegetable or an object?) and one to the same-category superordinate (e.g., Is it a four-legged animal, a bird or an insect?)], two concerned Subordinate Perceptual attributes (e.g., Has it got transparent wings, multicolored wings or no wings?) and the last two were about Subordinate Functional attributes (e.g., Does it jump, run or fly?). The probes were presented orally in standardised random order, first providing the name of the item and then asking the six questions, always in the same order. On this test BC made a total of 18 errors, which is at the lower limit of the normative group of healthy subjects [35].

In conclusion, all three patients showed a severe impairment of episodic anterograde memory. The qualitative profile of this impairment did not clearly differentiate psychogenic from organic amnesics. Indeed, all three patients performed poorly on free recall and recognition testing procedures involving both verbal and visual-spatial data. Performances on implicit memory tasks also failed to differentiate the two kinds of amnesics. In fact, all patients' performances were in the range of the normal controls on a repetition priming and on a skill learning task. BC's memory profile differed from that of PA and MC mainly for impairment of short-term and semantic memory. Moreover, as for retrograde memory for autobiographical events, BC's performances were poor for all of the periods examined. Instead, PA had substantially preserved memory for events that occurred 15 years or more before the onset of amnesia but decidedly poor memory for events that occurred in more recent periods.

## 5. Psychodiagnostic examination

At a clinical interview BC appeared collaborative but rather untroubled by her disability (*belle indifferance*). Her behaviour was childish and seductive, and she constantly tried to attract attention. The Rorschach test, administered and interpreted according to Rizzo's procedure [19], revealed an absence of thought disorders. The presence of an extroversive personality pro-

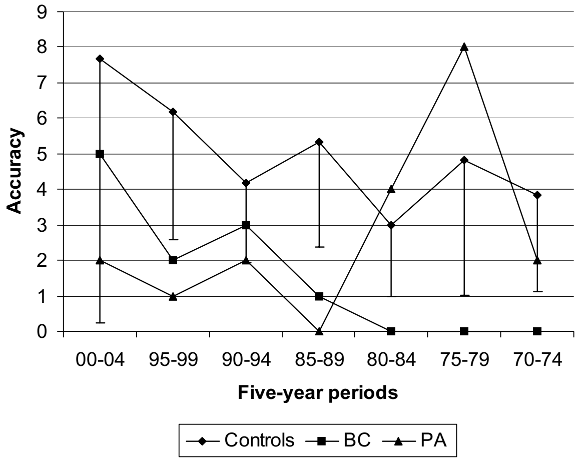

Fig. 2. Performance scores obtained by BC, PA and a group of 6 normal controls on Crovitz's procedure for assessing retrograde autobiographical memory as a function of the five-year periods examined.

file with reduced introspective ability revealed a seductive person, always looking for attention, with an insecure personality and traits of dependency. BC's affective expression was not very spontaneous and tended to be fleeting, with a high index of impulsiveness. Both the results of the clinical interview and the combination of the projective reactive indices revealed a fleeting and superficially affective expression, continuous searching for attention and the adoption of a seductive modality for establishing relations, suggesting that BC had a histrionic-hysterical personality profile.

At the clinical interview both PA and MC seemed to be fully aware of their disability and displayed moderately reactive depressive symptomatology. On the Rorschach test, PA's and MC's profiles were basically consistent with the organic syndrome described by Rorschach [5], with no evidence of psychiatric disease or personality disorders.

## 6. Discussion

In the present study we compared a patient with psychogenic amnesia with two patients affected by well-defined forms of organic amnesia to identify features that distinguish between these two kinds of memory disorder. For this purpose we considered four potentially useful aspects: *i*) the characteristics of the clinical appearance of the syndrome; *ii*) the congruence of cerebral damage and severity of the memory disorder; *iii*) the qualitative profile of the cognitive impairment as revealed by the neuropsychological assessment; *iiii*) the results of the psychopathological assessment.

One of the most peculiar and distinctive clinical signs of psychogenic amnesia is the loss of personal identity, i.e., when the patient fails to remember basic information about his own personal life and experiences a severe loss of knowledge and consciousness of self [14, 16,30,36,37,40]. In fact, immediately after the accident BC could not remember her name or address or the names of family members; years later she remembered this information but not as she had experienced it in her premorbid life. No similar loss of knowledge of self-identity was found in the clinical records of the two organic amnesics, even in the very early phases of their disease. Another, less frequently reported, distinctive sign of psychogenic amnesia is the loss of basic semantic knowledge and procedural skills [34]. In fact, in the period following the onset of amnesia BC had difficulty finding words in a conversation, and she had lost the ability to read and write; moreover, she was no longer able to take care of her children or do her housework (e.g. ironing, cooking). She only partially re-acquired these abilities many years after the onset of amnesia. Once again, in the clinical records of PA and MC there is no mention of a loss of basic linguistic or procedural skills.

In a patient with organic amnesia memory loss is usually the consequence of a severe pathological event (e.g. stroke, traumatic brain injury, anoxia-ischemia, encephalitis), and neuroradiological investigation invariably documents the presence of cerebral damage affecting the medial temporal lobe or the diencephalic nuclei or basal forebrain [44]. Instead, in the psychogenic form of amnesia a mild pathological event (e.g. a mild head trauma) typically precipitates the memory disorder [3,16,25,36,37,53] and neuroradiological investigation usually discloses no sign of cerebral sufferance [3, 16,22,38,40,53]. Consistent with this view, the pathological event that precipitated BC's amnesia was a very mild head trauma, and she did not present any neurological or neuroradiological sign of cerebral sufferance. In contrast, serious clinical events (i.e., prolonged hypoglycaemic coma or an expansive lesion of the basal diencephalon) were at the origin of the memory disorder in the organic amnesics. Further, neuroradiological examination revealed severe bilateral hippocampal atrophy in one patient and a focal lesion affecting the basal forebrain in the other.

A careful neuropsychological assessment is critical for differentiating between organic and psychogenic forms of amnesia. In the typical organic patient dense memory impairment is observed in the context of substantially spared general intellectual functioning. The memory deficit typically affects acquisition of events and facts encountered post-morbidly; thus, the patient inevitably fails to recall (and generally also to recognise) new information. Also, the retrieval of events acquired pre-morbidly is typically compromised in organic amnesics, and this retrograde memory deficit frequently (but not invariably) shows a temporal gradient in which recent memories are more severely affected than remote ones. Although organic amnesia is characterised by a pervasive and devastating loss of memory, some components of memory remain intact. So, these patients are normally able to retrieve over-learned semantic memories and to perform in the normal range on tasks of short-term and working memory; further, they show normal implicit memory on tasks of skill learning, classical conditioning and repetition priming [for reviews see 33 and 44]. The neuropsychological profile disclosed by PA and MC substantially conformed to the profile typically observed in organic amnesia. Indeed, both patients had high IQs and performed normally on tests investigating a variety of cognitive domains; the only exception was PA's lower than normal scores on tests of executive functions. Moreover, the two patients performed consistently below normal on tests of anterograde episodic memory but in the normal range on tests of short-term memory, semantic memory and verbal repetition priming. Finally, PA's temporal profile of retrograde memory impairment was reminding of a negative temporal gradient. In fact, the patient's memory loss was more severe for the 15 years preceding the onset of amnesia but affected much less memories acquired in the more remote period.

BC's cognitive profile was unusual with respect to that typically observed in organic amnesia. First, she did not present an isolated memory deficit. Indeed, she performed inconsistently on tests of general intelligence, with a pathological IQ on the WAIS-R and a normal score on Raven's Matrices. BC was only mildly impaired on tests of executive functioning and she scored normally on tests of language and visual-spatial abilities. BC's memory profile was particularly atypical for an organic origin of her amnesic syndrome. In line with the above-reported predictions, she had a severe retrograde memory impairment and, differently from PA, her loss of premorbid memories did not show any signs of a negative temporal gradient. Given the amount of time that had elapsed from BC's onset of amnesia (and to a lesser extent PA's), we could not exclude that some kind of relearning of retrograde events had occurred. Such a phenomenon, potentially able to influence the overall performance level and/or the temporal profile of remembered events in Crovitz's procedure, is obviously difficult to verify or to take into account in the performance analysis. BC was also severely impaired in anterograde memory tests. In this case, however, the qualitative analysis of the impairment did not reveal any peculiarity able to differentiate BC's performance from those of the two organic amnesics. In fact, her deficit encompassed both verbal and visual-spatial information and was equally severe when free recall or recognition testing procedures were used. In striking contrast with what is generally observed in organic amnesics (and confirmed in PA and MC), BC showed a deficit of verbal and spatial short-term memory and poor semantic memory. BC's performances were consistently normal only in the implicit memory domain; she demonstrated substantial priming in a Stem Completion paradigm and clear improvement of mirror reading skill with practice.

According to the literature, a psychopathological condition is frequent in the clinical records of patients with a psychogenic form of amnesia. It may consist of a premorbid history of psychiatric disease (e.g., depression) [25,30,36], emotional stress immediately preceding the onset of the amnesic syndrome (e.g., marital trouble) [22,25,30,36,38,40] or a histrionic-hysterical personality profile, documented by psychopathological assessment at the time of the experimental investigation [3,28]. A thorough anamnestic investigation of BC failed to document a history of depression or other psychiatric diseases or significant emotional stress in the period preceding the onset of the amnesic syndrome. However, both the clinical interview and the psychodiagnostic evaluation documented that BC's personality profile was characterised by histrionic-hysterical traits. The same psychodiagnostic reactive, administered to PA and MC, revealed a mild depressive state congruent with disability due to their amnesic state.

In summary, the results of the present study outline four areas of clinical and instrumental investigations that clearly distinguish between organic and psychogenic forms of amnesia. Briefly, organic amnesia is recognised when $i$) the memory disorder spares personal identity, basic semantic knowledge and procedural skills; *ii*) the pathological event precipitating the syndrome and the neuroradiological investigation are consistent with cerebral damage affecting cortical and/or subcortical areas known to be critical for declarative long-term memory; *iii*) the neuropsychological investigation documents an impairment of declarative episodic memory that contrasts with a substantial sparing of other memory functions and general intelligence;

*iiii*) no relevant predisposing psychiatric conditions can be found in the premorbid history or in the current investigation. In contrast, a memory disorder is very likely psychogenic when $i$) the amnesic syndrome, at least in the very early phases, includes the loss of personal identity and very basic semantic and procedural abilities; *ii*) damage directly affecting cerebral areas critical for memory functioning can not be suspected based on clinical history or neuroradiological exams; *iii*) the neuropsychological examination reveals a general intellectual dysfunction and/or a qualitative profile of memory disorder inconsistent with that typically observed in organic amnesia; *iiii*) a psychopathological condition is revealed in the premorbid history and/or in the current psychodiagnostic examination. Obviously, the four differential criteria are not always so clearly fulfilled as in the present cases, and the relative importance of each of the four criteria may change depending on the individual case. For example, the lack of any neuroradiological and perfusional evidence of cerebral sufferance is immediately suggestive of a psychogenic form of amnesia. However, in a patient with a neuroradiologically documented lesion in the temporal and/or frontal lobes but in whom psychogenic amnesia is suspected, the discriminative role of data deriving from clinical, neuropsychological and psychodiagnostic examinations is crucial. On the contrary, in a patient with an unusual neuropsychological profile of memory impairment and with anamnestic or current evidence of psychiatric disease, evidence of cerebral damage directly affecting structures in the Papez circuit always indicates that the memory disorder is organic in origin.

Finally, there are two other conditions, characterised by significant memory disorder, that can not be regarded as either organic or psychogenic. The first is so-called “functional amnesia”. This term, coined by De Renzi et al. [16], refers to patients in whom a pervasive anterograde/retrograde memory impairment is not associated with significant brain damage but in whom it is impossible to identify significant psychopathological conditions underlying the memory disorder. In the few reported cases in which the term “functional amnesia” was not a tantamount of “psychogenic amnesia” [15, 16,50] the impairment of retrograde memory, involving both autobiographical and public events memories, was the most severe cognitive deficit. Sometimes retrograde amnesia was associated with a mild anterograde deficit [50] or with a deficit of semantic or procedural memory [16,50]. Sense of personal identity was compromised in two cases [15,16].

Memory disorders are the cognitive deficits most typically malingered by subjects who try to obtain financial benefits from a traumatic event. There is a great deal of literature on neuropsychological and psychodiagnostic tools for discovering simulation in a patient complaining of memory impairment. In the case of BC, malingering could be excluded based on the duration of the patient’s clinical history (8 years at the time of our assessment), the lack of evident financial benefits following the onset of the memory disorder and, finally, based on the fact that the patient and her relatives were actively searching for a remedy for her disability due to memory disorder.

## 7. Conclusions

Psychogenic amnesia is a complex disorder characterised by a wide variety of symptoms. Consequently, in a number of cases it is difficult to distinguish it from organic memory impairment. Our study points out the usefulness of a multidimensional evaluation of clinical, neuroradiological, neuropsychological and psychopathological aspects to provide convergent findings useful for differentiating between the two forms of memory disorder.
